# Laparoscopic Surgery Can Reduce Postoperative Edema Compared with Open Surgery

**DOI:** 10.1155/2016/5264089

**Published:** 2016-09-29

**Authors:** Dong Guo, Jianfeng Gong, Lei Cao, Yao Wei, Zhen Guo, Weiming Zhu

**Affiliations:** Department of General Surgery, Jinling Hospital, Medical School of Nanjing University, No. 305 East Zhongshan Road, Nanjing 210002, China

## Abstract

*Aim*. The study aimed to investigate the impact of laparoscopic surgery and open surgery on postoperative edema in Crohn's disease.* Methods*. Patients who required enterectomy were divided into open group (Group O) and laparoscopic group (Group L). Edema was measured using bioelectrical impedance analysis preoperatively (PRE) and on postoperative day 3 (POD3) and postoperative day 5 (POD5). The postoperative edema was divided into* slight edema* and* edema *by an edema index, defined as the ratio of total extracellular water to total body water.* Results*. Patients who underwent laparoscopic surgery had better clinical outcomes and lower levels of inflammatory and stress markers. A total of 31 patients (26.05%) developed* slight edema* and 53 patients (44.54%) developed* edema* on POD3. More patients developed postoperative edema in Group O than in Group L on POD3 (*p* = 0.006). The value of the edema index of Group O was higher than that of Group L on POD3 and POD5 (0.402 ± 0.010 versus 0.397 ± 0.008, *p* = 0.001; 0.401 ± 0.009 versus 0.395 ± 0.007, *p* = 0.039, resp.).* Conclusions*. Compared with open surgery, laparoscopic surgery can reduce postoperative edema, which may contribute to the better outcomes of laparoscopic surgery over open surgery.

## 1. Introduction

As a minimally invasive surgery, laparoscopic surgery brings many short-term and long-term benefits over open surgery, such as reducing postoperative complications, promoting postoperative recovery, and reducing hospital stay [1, 2]. The benefits of laparoscopic surgery over open surgery are associated with reducing surgical trauma and stress, but the precise mechanism is still unclear [3, 4].

Surgical trauma and stress can lead to a multitude of systemic responses, which can be reflected by the levels of inflammatory and stress markers such as cytokines, acute phase proteins, and stress hormones [4–6]. The response increases the permeability of the capillary membranes and affects fluid redistribution between intravascular and interstitial space, which can lead to local and general edema postoperatively [7–9]. Previous studies showed that postoperative edema is associated with poor clinical outcomes [7, 8]. We hypothesized that when compared with open surgery, laparoscopic surgery can alleviate postoperative edema by reducing surgical trauma and lower level of systemic response, which may contribute to better outcomes of laparoscopic surgery.

Crohn's disease (CD) is a chronic inflammatory gastrointestinal disorder, characterized by phases of remission and frequent relapses that often need surgical intervention [10]. Surgery is often necessary to treat complications such as stricture, fistula, abscess, bleeding, or failed responses to medical therapy [10, 11]. Laparoscopic surgery has been widely applied in patients with CD because its safety and feasibility were confirmed, and many studies have exhibited its advantages over open surgery [12–14]. However, there are no reports about postoperative edema in patients with CD.

Bioelectrical impedance analysis (BIA) is widely used to measure body water to assess body water, find nonclinically evident edema, and manage fluid [15–18]. Compared with subjective methods, BIA is a more objective, numerical, and credible method to assess body water and edema, particularly for nonclinically evident edema [15]. The edema index, defined as the ratio of extracellular water to total body water, can present the level of whole body edema and segmental edema. According to the edema index, edema can be graded to* slight edema* (defined as edema index ≥ 0.395) and* edema* (defined as edema index ≥ 0.400) [19]. Segmental edema includes five segments as follows: right arm, left arm, trunk, right leg, and left leg [20].

The aim of this study is to investigate the postoperative edema in CD and compare the impact of laparoscopic and open surgery on postoperative edema in a single disease.

## 2. Materials and Methods

### 2.1. Patient Enrollment and Grouping

From January 2013 to October 2015, a series of patients with CD were enrolled in this study at the Inflammatory Bowel Disease Center of Jinling Hospital, Nanjing, China. All patients required enterectomy with indications, including uncontrolled inflammation, stricture/mass, internal fistula, and hemorrhage. The strict exclusion criteria included enterocutaneous fistula, abscess, vaginal fistula, extensive abdominal adhesions, and any diseases that could influence water distribution, such as hypertension, renal disease, liver dysfunction, endocrine disorder, or other systemic diseases. All patients were in fine preoperative physical condition with American Society of Anesthesiologists (ASA) classification I or II. All of those selective operations can be performed by either open or laparoscopic procedure evaluated by the whole team. According to patients' choice, patients were divided into two groups as follows: open surgery (Group O) and laparoscopic surgery (Group L). This prospective, observational cohort study was approved by the Institutional Review Board in Jinling Hospital and complied with the Declaration of Helsinki. All patients provided written informed consent.

### 2.2. Data Collection

Blood was sampled and analyzed on the day before surgery (PRE) and on postoperative day 1 (POD1), postoperative day 3 (POD3), and postoperative day 5 (POD5) at Central Clinical Laboratory in Jinling Hospital. Body water was analyzed preoperatively (PRE) and on POD3 and POD5 using BIA (InBody 3.0, Biospace, Korea) as previously described [21]. Patients' baseline characteristics, perioperative data, inflammatory and stress markers, fluid management, complications and 1-month follow-up, were recorded prospectively.

### 2.3. Sample Size Calculation

Our preliminary experiment showed that 60% of patients (6/10) in Group O and 30% of patients (3/10) in Group L developed edema on POD3, respectively. Accordingly, it was determined that 42 patients per group were needed, based on alpha of 0.05 with a power of 80%. Considering that patient may drop out, a total sample size of at least 86 patients would be included. More patients would be enrolled if necessary.

### 2.4. Laparoscopic and Open Procedure

All surgeries, including laparoscopic surgery and open surgery, were performed by one group, including two experienced senior surgeons (who are experts in gastrointestinal surgery), two junior surgeons, and two residents.

In laparoscopic procedures, the carbon dioxide pneumoperitoneum pressure was kept at 12 mmHg. Stapled anastomose was constructed in a side-to-side fashion using a 60 mm linear stapler (ECHELON FLEX Ethicon Endo-Surgery LLC, Guaynabo, USA). After resection and stapled anastomosis, we regularly reinforced the anastomosis with absorbable sutures. Generally, the surgery was finished in a totally laparoscopic procedure. Hand-assisted anastomosis was needed to make ensure the quality of anastomosis, when the risk of laparoscopic anastomosis was high.

In open procedures, we had a side-to-side anastomosis using a 75 mm linear cutter stapler (PROXIMATE, Ethicon Endo-Surgery LLC, Guaynabo, USA), and we reinforced the anastomosis with absorbable sutures routinely.

### 2.5. Fluid Management

All patients received adjusted, restricted, perioperative fluid administration according to the concept of “fast track” treatment, as previously described in our department [22]. Postoperative fluid management was not inflexible but was decided by senior clinicians according to integrated clinical consideration including heart rate, blood pressure, urine output, and serum lactate. Intravenous fluid was discontinued when patients could tolerate oral drink or enteral nutrition feeding well.

### 2.6. Postoperative Management

Postoperative management was performed according to the “fast track rehabilitation program” in our department [23]. Postoperative pain was managed by patient-controlled analgesia for both groups. Patients were mobilized within the first 6–12 h after surgery. Urinary catheters were removed on POD1. If the patient tolerated it well, a nasogastric feeding tube replaced the nasogastric drainage tube for postoperative liquid and enteral nutrition feeding by a feeding pump. The patients were fed with clear liquid on POD1 and enteral nutrition on POD2 depending on patients' situations. The time to tolerate enteral nutrition of approximately 15 to 20 kcal/kg/day was recorded. Patients were discharged when enteral nutrition was well tolerated (approximately 25 to 30 kcal/kg per day) without postoperative complications that required surgical interventions.

### 2.7. Statistical Analysis

Statistical analysis was performed using SPSS version 18.0 software (SPSS Inc., Chicago, IL, USA). Quantitative variables, presented as mean ± standard deviation (SD), were analyzed by Mann-Whitney* U* test, Student's* t*–test, or Analysis of Variance (ANOVA) followed by post hoc test if appropriate. Quantitative variables, expressed as a number (percentage), were analyzed by Pearson's *χ* ^2^ test or Fisher's exact test. A *p* < 0.05 was considered statistically significant.

## 3. Results

### 3.1. Patients' Baseline Characteristics

A total of 131 patients were enrolled in this study, and 12 patients were excluded during surgery: seven patients in Group O were excluded because of severe abdominal adhesion during surgery, and five patients in Group L were excluded for the conversion into an open procedure at the start of surgery. Overall, 119 patients finished the whole study, with 61 patients in Group O and 58 patients in Group L. All patients underwent routine enterectomy, and no deaths were reported. All patients were discharged without any complications that required surgical interventions.

There was no significant difference between groups in terms of demographic and baseline characteristics (Table 1). The clinical characteristics including Crohn's Disease Activity Index (CDAI), duration of disease, Montreal Classification, and ASA classification were well matched between groups. No significant difference was found in drug, smoking, and operation histories. After sufficient preoperative nutritional support, nutritional status was improved, and no patient had severe hypoalbuminemia or anemia.

There was no difference in surgical indications (*p* = 0.827) and types of surgery (*p* = 0.545) between groups (Table 2). Sixteen patients (27.59%) have hand-assisted anastomosis during the laparoscopic procedure. The length of incision of Group O was much longer than that of Group L (10.54 ± 1.91 versus 5.24 ± 1.39 cm, *p* < 0.001), and intraoperative blood loss was less in Group L (128.36 ± 55.05 versus 57.24 ± 33.88 mL, *p* < 0.001). The duration of operations for laparoscopic procedures was approximately 1.3 times as much as for open procedures (119.29 ± 30.76 versus 91.70 ± 23.77 min, *p* < 0.001).

### 3.2. Fluid Intake and Output

**Figure 1:**
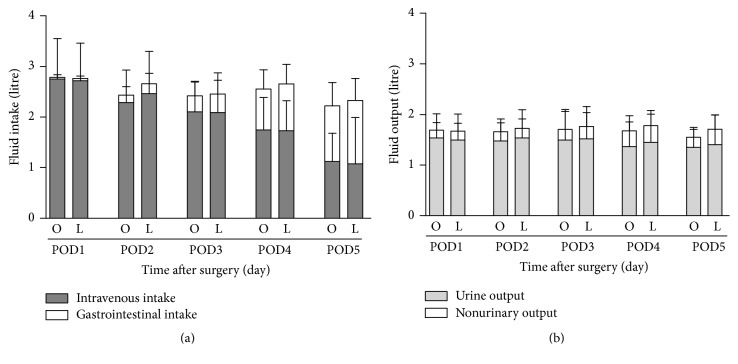
Postoperative fluid management. O: Group O; L: Group L. POD: postoperative day. (a) Daily volume of fluid intake after surgery including intravenous fluid intake and gastrointestinal fluid intake. (b) Daily volume of fluid output after surgery including urine output and nonurinary output. “Nonurinary output” included digestive fluid, drainage fluid, and feces. There was no difference between groups.

The fluid intake included intravenous intake and gastrointestinal intake, and fluid output included urine and no urinary output, such as digestive fluid, drainage fluid, and feces. Each day's volumes of fluid intake and output were similar between groups (Figure 1). The total volumes of fluid intake and output were higher in Group O than in Group L in the perioperative period (12.422 ± 0.203 versus 12.866 ± 0.262 L, *p* < 0.001; 8.290 ± 0.221 versus 8.652 ± 0.321 L, *p* < 0.001).

### 3.3. Clinical Outcomes

Patients had better postoperative outcomes after laparoscopic procedures than after open procedures (Table 3). The time to flatus was shorter in Group L than in Group O (41.40 ± 11.97 versus 51.80 ± 14.94 hours, *p* < 0.001), as well as time to bowel movement (64.84 ± 19.44 versus 75.14 ± 22.95 hours, *p* = 0.004). Most patients tolerated enteral nutrition of approximately 15 kcal/kg per day on POD4 or POD5, and the time of group L was earlier than that of group O (4.41 ± 1.26 versus 5.00 ± 1.43 days, *p* = 0.013). Although patients who underwent laparoscopic procedures had higher surgical cost (*p* < 0.001), the total cost was similar (*p* = 0.602). Laparoscopic surgery can shorten the hospital stay by approximately 1 day (7.62 ± 2.86 versus 8.64 ± 3.52 days, *p* = 0.041).

Surgery-associated complications for CD included incision-associated complications (infection and dehiscence), anastomotic leakage, and intra-abdominal abscess or mass and other complications (Table 3). Fewer patients had complications in Group L than in Group O but without statistical difference (19 patients [31.15%] versus 15 patients [25.86%], *p* = 0.524).

### 3.4. Inflammatory and Stress Markers

**Figure 2:**
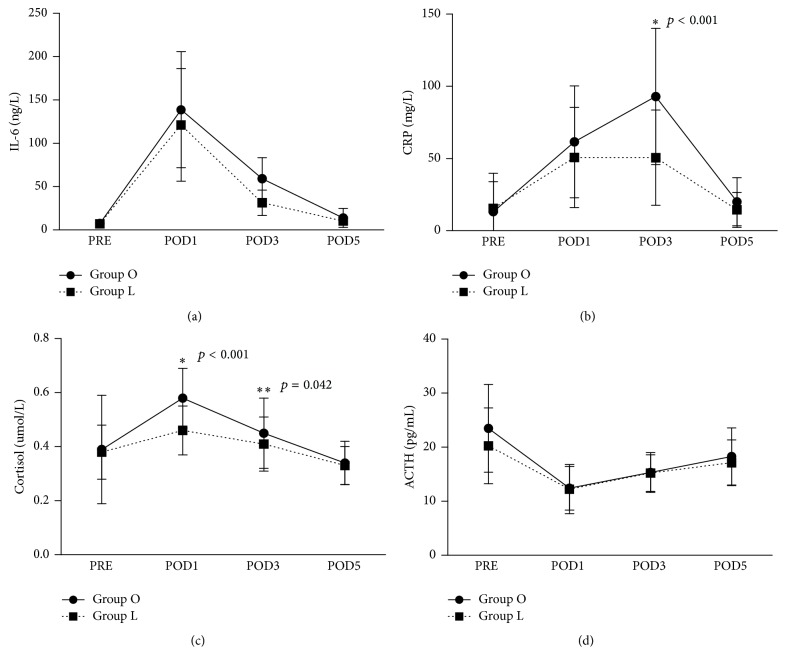
Evolution of inflammatory and stress markers levels. The *p* values were calculated between Group O and Group L using* t*-test or Mann-Whitney* U* test. Asterisks *∗* and *∗∗* indicate statistical significance, and the *p* values are shown. PRE: preoperatively; POD: postoperative day.

The evolution of serum inflammatory and stress marker levels is shown in Figure 2. For all patients, the level of interleukin-6 (IL-6) for PRE was lower than that on POD1 (7.28 ± 4.36 versus 130.57 ± 67, *p* < 0.001) and POD3 (7.28 ± 4.36 versus 46.15 ± 24.41, *p* < 0.001). However, the level of IL-6 for PRE was similar to that on POD5 (7.28  ±  4.36 versus 12.08  ±  10.98, *p* < 0.001). The same tendency was found for C-reactive protein (CRP) and cortisol as well. The level of adrenocorticotropin hormone (ACTH) for PER was higher than that of POD1 and POD3 (21.99 ± 8.09 versus 12.34 ± 4.03, *p* < 0.001; 21.99 ± 8.09 versus 15.26 ± 3.67, *p* = 0.013, resp.), but similar to that on POD5 (21.99 ± 8.09 versus 17.74 ± 5.26, *p* = 0.221).

When comparing the groups, baseline levels of IL-6, CRP, cortisol, and ATCH were similar (*p* = 0.994, *p* = 0.50, *p* = 0.821, and *p* = 0.275, resp.). The levels of all markers were higher in Group O than in Group L. The levels of CRP in Group O were significantly higher than in Group L (*p* < 0.001) on POD3. The level of cortisol was significantly higher in Group O than in Group L on POD3 and POD5 (*p* < 0.001 and *p* = 0.042, resp.).

### 3.5. Body Water Analysis

The edema index changed greatly after surgery for both groups and two-way ANONV was used to examine the change of edema before and after surgery. The value of edema index on POD3 and POD5 was significantly higher than that preoperatively (0.399 ± 0.016 versus 0.388 ± 0.012, *p* < 0.001; 0.395  ±  0.010 versus 0.388  ±  0.012, *p* = 0.011, resp.); there was no difference between POD3 and POD5 (0.399 ± 0.016 versus 0.394 ± 0.010, *p* = 0.12). The impact of laparoscopic and open surgery on postoperative edema was compared in the following aspects.

First, the grades of edema are listed in Table 4. A total of 31 patients (26.05%) developed* slight edema*, and 53 patients (44.54%) developed* edema *on POD3; there were 41 patients (34.45%) with* slight edema* and 32 patients (26.89%) with* edema* on POD5. There was no difference in edema grades between groups preoperatively. More patients developed* slight edema* and* edema *in Group O than in Group L on POD3 (*p* = 0.006), but not on POD5 (*p* = 0.145).

Second, the values of the edema index are listed in Table 5. The edema index of most patients increased after surgery. The value of the edema index of Group O was significantly higher than that of Group L on POD3 and POD5 (0.402  ±  0.010 versus 0.397 ± 0.008, *p* = 0.001; 0.401 ± 0.009 versus 0.395 ± 0.007, *p* = 0.039, resp.). The increment of the edema index (Δ) was defined as the difference in values of the edema index (Δ3 = POD3-PRE; Δ5 = POD5-PRE). The increment of the edema index was significantly greater in Group O than in Group L on POD3 (Δ3: 0.015 ± 0.015 versus 0.007 ± 0.009, *p* = 0.002), but not significantly different on POD5 (Δ5: 0.009 ± 0.016 versus 0.005 ± 0.012, *p* = 0.383).

Third, the segmental edema is shown in Table 5. The five segmental edema indexes increased to a certain extent in both groups after surgery, with a remarkable difference between groups in the right arm on POD3 (0.384 ± 0.009 versus 0.379 ± 0.012, *p* = 0.013). The increment of the segmental edema index (Δ^s^) was higher in Group O than in Group L. The Δ^s^ of trunk, right leg, and left leg in Group O was significantly higher than that of Group L (0.014 ± 0.015 versus 0.007 ± 0.013, *p* = 0.020; 0.014 ± 0.015 versus 0.007 ± 0.013, *p* = 0.020; 0.013 ± 0.018 versus 0.008 ± 0.012, *p* = 0.045, resp.).

## 4. Discussion

To our knowledge, our study is the first to report the incidence of postoperative edema in CD after enterectomy. The results have shown that the edema index increased significantly after surgery and there was approximately 71% edema, including* slight edema *and* edema*, after surgery. It was reported that about 53% (20/38) of patients develop edema after major abdominal surgery [7] and Vaughan-Shaw et al. reported that approximately 35% (19/55) of patients develop edema after emergency abdominal surgery [8]. The different incidences may be ascribed to different methods used to assess edema and the heterogeneity of patients enrolled; in the two studies, the patients had different primary diseases, physical conditions, and nutritional status.

The impact of laparoscopic surgery and open surgery on postoperative edema was compared from different aspects, as described above. A smaller number of patients with postoperative edema and lower value and increment of the edema index were found in the laparoscopic surgery group than the open surgery group. Perioperative fluid management, nutritional status, different surgery, and systemic diseases have great influences on postoperative edema [24, 25]. In the present study, we excluded those interfering factors as much as possible. All patients were in fine preoperative physical condition, including the nutritional status. Both groups adopted the same fluid infusion strategy, and each day's volume was not different in the perioperative period. The total volume of fluid intake and output was higher in the laparoscopic surgery group than in the open surgery group. Fluid management was applied according to body weight, and if fluid infusion affected the postoperative fluid redistribution, more edema would have been found in laparoscopic surgery rather than in open surgery. Overall, we believe that it was the different surgery that influences postoperative edema, and laparoscopic surgery can reduce postoperative edema when compared with open surgery.

When compared with conventional open surgery, the benefits of laparoscopic surgery have been widely investigated and confirmed in CD [12, 13, 26–29]. The present study demonstrated those benefits as well, including reducing intraoperative blood loss and length of incision, speeding postoperative recovery, shortening hospital stay, and reducing surgical-related complications, especially incision-associated complications. Meanwhile, there were still disadvantages for laparoscopic surgery in CD [27], such as requiring experienced laparoscopic surgeons and skills, increased cost and time, and not being suited for patients with severe complications and intra-abdominal adhesions.

Unlike local edema caused by local surgery, such as thyroidectomy or hand surgery [7, 8, 30], all five segmental edema indexes increased after surgery, indicating that abdominal surgery resulted in generalized edema. The generalized edema is associated with a systemic response to surgery [4, 31, 32]. Surgical trauma and stress can lead to a multitude of systemic responses, which encompass a wide range of interlinked endocrinological, metabolic, and immunological pathways [3–6, 14, 31, 32]. Through varied pathways and mediators, the response to surgical trauma can increase the permeability of the capillary membrane and results in a redistribution of plasma proteins and fluid from the intravascular to the interstitial space [7, 33]. Less postoperative edema indicated less surgical trauma and stress of laparoscopic surgery. In the perioperative period, the levels of inflammatory and stress markers and edema index increased and decreased, with a “peak value” on POD3, indicating the natural course of stress responses and body recovery after surgery.

Postoperative edema is associated with poor clinical outcomes, such as delayed healing, more complications, slow bowel function recovery, and longer hospital stay [7, 30]. Itobi et al. reported that postoperative edema could independently predict gastrointestinal recovery, and measurement of edema can be used to identify those patients at risk of poor clinical outcomes [7]. In an animal study, when compared with open surgery, the laparoscopic surgery groups had faster intestinal transit recovery, and the faster intestinal transit recovery was associated with less edematous changes [34]. The benefits of laparoscopic surgery are associated with less postoperative edema, surgical trauma, and stress to surgery accordingly [4–6, 31]. Overall, the present study suggested that laparoscopic surgery can reduce postoperative edema and response to surgical trauma and stress, as well as speed postoperative recovery compared with open surgery. Reduction of postoperative edema may elucidate the association of laparoscopic surgery with better clinical outcomes.

However, there were limitations in the present study. First, we did not observe the evolution of body water consecutively. Most patients were with drainage tubes, electrocardiograph monitoring, and bellybands, which interfere with the result of BIA on POD1 and POD2. Second, it was not a random study. We tried to randomize patients to different surgery procedures in the same ward, but it failed. Instead, we adopted strict inclusion and exclusion criteria to reduce bias as much as possible, and there no difference in preoperative characteristics between the groups. Further randomized clinical trials are required.

## 5. Conclusions

We reported for the first time incidence of postoperative edema in CD. Compared with open surgery, laparoscopic surgery can reduce postoperative edema and speed postoperative recovery and reduce levels of inflammatory and stress responses to surgery for patients with CD. Alleviation of postoperative edema may contribute to enhanced recovery after laparoscopic surgery compared to open surgery.

## Figures and Tables

**Table 1:** The demographic and baseline characteristics of patients.

	Group O (*n* = 61)	Group L (*n* = 58)	*p* values^a^
Age (years)	33.39 ± 12.21	31.19 ± 11.02	0.380^b^
Sex (female/male)	21/40	19/39	0.847
CDAI	143.87 ± 54.76	146.57 ± 63.48	0.869^b^
Duration of disease (months)	49.56 ± 41.22	48.84 ± 37.60	0.869^b^
History of steroid usage	32 (52.46)	29 (50.00)	0.789
History of infliximab	9 (14.75)	11 (18.97)	0.539
History of smoking			0.923
Never	36 (59.02)	34 (58.62)	
Former	25 (40.98)	24 (41.38)	
Montreal classification			
Age of diagnosis			0.760
A1 ≤16 years	5 (8.20)	6 (10.34)	
A2 17–40 years	39 (63.93)	39 (67.24)	
A3 >40 years	17 (27.87)	13 (22.41)	
Disease location			0.931
L1 (ileum)	18 (29.51)	17 (29.31)	
L2 (colonic)	11 (18.03)	12 (20.69)	
L3 (ileocolonic)	32 (52.46)	29 (50.00)	
Disease behavior			0.749
B1 (nonstrict.nonfist)	8 (13.11)	7 (12.07)	
B2 (stricturing)	36 (59.02)	38 (65.52)	
B3 (fistulizing)	17 (27.87)	13 (22.41)	
Perianal disease	18 (29.51)	14 (24.14)	0.509
History of appendectomy	17 (27.87 )	15 (25.86)	0.805
Previous abdominal operations			0.758
0	38 (62.30)	39 (67.24)	
1	18 (29.51)	16 (27.59)	
2	5 (8.20)	3 (5.17)	
ASA			0.537
I	41 (67.21)	42 (72.41)	
II	20 (32.79)	16 (27.59)	
Nutritional status			
BMI (kg/m^2^)	18.49 ± 2.53	18.58 ± 2.87	0.857^b^
Albumin (g/L)	37.58 ± 4.07	37.39 ± 4.04	0.811^b^
Total protein (g/L)	62.39 ± 7.84	63.37 ± 7.71	0.896^b^
Hemoglobin (g/L)	113.79 ± 17.45	114.29 ± 15.95	0.869^c^
Soft lean mass (kg)	40.82 ± 9.38	40.88 ± 8.69	0.994^c^
Skeletal muscle mass (kg)	24.13 ± 5.78	25.14 ± 5.86	0.796^c^

Data are *n* (percentages) and mean ± SD. Group O: patients who underwent open surgery; Group L: patients who underwent laparoscopic surgery; CDAI: Crohn's Disease Activity Index; ASA: American Society of Anesthesiologists; BMI: Body Mass Index.

^a^Chi-square test, except ^b^Mann-Whitney *U* test, and ^c^ *t*-test.

**Table 2:** Comparison of intraoperative data between groups.

	Group O (*n* = 40)	Group L (*n* = 39)	*p* values^a^
Surgical indication			0.968^b^
Inflammation	3 (7.50)	3 (7.69)	
Stricture	24 (60.00)	23 (58.97)	
Internal fistula/mass	11 (27.50)	10 (25.64)	
Hemorrhage	2 (5.00)	3 (7.69)	
Type of surgery			0.629^b^
Small bowel resection	11 (27.50)	10 (25.64)	
Ileocolic resection	21 (52.50)	24 (61.54)	
Colonic resection	8 (20.00)	5 (12.82)	
Hand-assisted anastomosis		11 (28.21)	
Length of incision (cm)	10.30 ± 1.79	5.00 ± 1.23	<0.001
Operation duration (min)	86.85 ± 24.31	121.53 ± 33.62	<0.001
Blood loss (mL)	122.25 ± 53.61	54.10 ± 21.97	<0.001

Data are *n* (percentages) and mean ± SD. Group O: patients who underwent open surgery; Group L: patients who underwent laparoscopic surgery.

^a^Mann-Whitney *U* test except ^b^Chi-square test.

**Table 3:** Comparison of clinical outcomes between groups.

	Group O (*n* = 61)	Group L (*n* = 58)	*p* values^a^
Postoperative recovery			
Time to flatus (hours)	51.80 ± 14.94	41.40 ± 11.97	<0.001
Time to bowel movement (hours)	75.14 ± 22.95	64.84 ± 19.44	0.004
Time to tolerate EN (days)	5.00 ± 1.43	4.41 ± 1.26	0.013
Hospital stay and cost			
Postoperative stay (days)	8.64 ± 3.52	7.62 ± 2.86	0.041
Total cost (USD)	8945.82 ± 2365.90	9114.74 ± 2266.18	0.602
Surgical cost (USD)	844.00 ± 253.85	1132.71 ± 235.63	<0.001
Complications	19 (31.15)	15 (25.86)	0.524^b^
Infection of incision	8 (13.11)	4 (6.90)	
Dehiscence of incision	2 (3.28)	1 (1.72)	
Anastomotic leakage	2 (3.28)	1 (1.72)	
Abscess/mass	4 (6.56)	5 (8.62)	
Others^c^	3 (4.92)	4 (6.90)	

Data are *n* (percentages) and mean ± SD. Group O: patients who underwent open surgery; Group L: patients who underwent laparoscopic surgery. EN: enteral nutrition.

^a^Mann-Whitney *U* test except ^b^Chi-square test. ^c^Others include urinary tract infection, throat infection, and catheter-related infection.

**Table 4:** Comparison of evolution of edema grades between groups.

	Group O (*n* = 61)	Group L (*n* = 58)	*p* values
PRE			0.428
Normal	45 (73.77)	43 (74.14)	
Slight edema	13 (21.31)	9 (15.52)	
Edema	3 (4.92)	6 (10.34)	
POD3			0.006
Normal	10 (16.39)	25 (43.10)	
Slight edema	19 (31.15)	12 (20.69)	
Edema	32 (52.46)	21 (36.21)	
POD5			0.145
Normal	20 (32.79)	26 (44.83)	
Slight edema	20 (32.79)	21 (36.21)	
Edema	21 (34.43)	11 (18.97)	

Data are *n* (percentages). Group O: patients who underwent open surgery; Group L: patients who underwent laparoscopic surgery; PRE: preoperatively. POD3: postoperative day 3. POD5: postoperative day 5. *p* values by Chi-square test.

**Table 5:** The evolution of edema index and segmental edema index between groups.

	Group O (*n* = 61)	Group L (*n* = 58)	*p* values^a^
Whole body edema			
PRE	0.388 ± 0.015	0.389 ± 0.008	0.723
POD3	0.402 ± 0.010	0.397 ± 0.008	0.001^b^
POD5	0.397 ± 0.007	0.394 ± 0.007	0.039
Δ3	0.015 ± 0.015	0.007 ± 0.009	0.002
Δ5	0.009 ± 0.016	0.005 ± 0.012	0.383
Segmental edema			
Right arm			
PRE	0.374 ± 0.017	0.375 ± 0.011	0.377
POD3	0.384 ± 0.009	0.379 ± 0.012	0.013^b^
POD5	0.376 ± 0.006	0.376 ± 0.007	0.994^b^
Δ^s^3	0.010 ± 0.018	0.004 ± 0.015	0.238
Δ^s^5	0.004 ± 0.019	0.003 ± 0.011	0.491
Left arm			
PRE	0.374 ± 0.009	0.376 ± 0.010	0.479
POD3	0.384 ± 0.009	0.381 ± 0.012	0.226^b^
POD5	0.376 ± 0.009	0.378 ± 0.008	0.581
Δ^s^3	0.009 ± 0.012	0.006 ± 0.012	0.114^b^
Δ^s^5	0.004 ± 0.012	0.004 ± 0.011	0.988^b^
Trunk			
PRE	0.388 ± 0.012	0.391 ± 0.010	0.181^b^
POD3	0.402 ± 0.013	0.398 ± 0.012	0.107
POD5	0.396 ± 0.008	0.393 ± 0.009	0.284
Δ^s^3	0.014 ± 0.015	0.007 ± 0.013	0.020
Δ^s^5	0.006 ± 0.010	0.005 ± 0.011	0.492^b^
Right leg			
PRE	0.388 ± 0.012	0.391 ± 0.010	0.181^b^
POD3	0.402 ± 0.013	0.398 ± 0.012	0.107
POD5	0.396 ± 0.008	0.393 ± 0.009	0.284
Δ^s^3	0.014 ± 0.015	0.007 ± 0.013	0.020
Δ^s^5	0.006 ± 0.010	0.005 ± 0.011	0.492^b^
Left leg			
PRE	0.391 ± 0.015	0.394 ± 0.010	0.275
POD3	0.404 ± 0.013	0.402 ± 0.008	0.269^b^
POD5	0.399 ± 0.008	0.396 ± 0.014	0.316
Δ^s^3	0.013 ± 0.018	0.008 ± 0.012	0.045
Δ^s^5	0.006 ± 0.011	0.004 ± 0.018	0.971

Data are mean ± SD. Group O: patients who underwent open surgery; Group L: patients who underwent laparoscopic surgery. PRE: preoperatively. POD3: postoperative day 3. POD5: postoperative day 5. Δ: difference values of edema index; Δ^s^: difference values of segmental edema index; Δ3: POD3-PRE. Δ5: POD5-PRE.

^a^Mann-Whitney *U* test except ^b^ *t*-test.
